# Diet and Food Allergy as Risk Factors for Asthma in the Arabian Gulf Region: Current Evidence and Future Research Needs

**DOI:** 10.3390/ijerph16203852

**Published:** 2019-10-12

**Authors:** Naser A. Alsharairi

**Affiliations:** Heart, Mind & Body Research Group, Menzies Health Institute Queensland, Griffith University, Gold Coast 4222, Australia; naser.alsharairi@gmail.com

**Keywords:** diet, food allergy, asthma, Arabian Gulf region

## Abstract

Asthma is a chronic respiratory disease which is associated with higher levels of systemic inflammation. The causes of asthma remain poorly understood. Unhealthy diet and food allergy are potential risk factors for developing asthma. The prevalence of asthma in the Arabian Gulf region (AGR), and Kuwait, Saudi Arabia and Qatar in particular, is higher than in other Eastern Mediterranean countries. In the AGR, diets tend to be of low nutritional value due to high levels of total energy, cholesterol, sodium, added sugars and saturated fat, and low levels of fiber, fruit and vegetables. A few studies that include children and adults in the AGR have suggested a potential link between unhealthy diets/specific food allergens and increased risk of asthma, however, the association of food allergy with asthma is still a controversial issue. The aim of this commentary is to consider the evidence from the AGR regarding the effects of diet/food allergy on asthma risk that may be used to make recommendations for future research.

## 1. Introduction

Asthma is a major cause of death and is associated with emerging comorbidities in adults such as diabetes, cancer, metabolic syndrome, cardiovascular diseases and mental disorders [1]. Food allergy represents an abnormal reaction of the gastrointestinal mucosal immune system to ingested food protein antigens passing the oral route [2]. Food allergy has been classified into immunoglobulin E (IgE) mediated allergy and non-IgE mediated allergy.IgE-mediated allergy has an early-onset starting within two hours of eating allergenic foods, and causes common skin and gastrointestinal tract symptoms and evidence of circulatory or respiratory compromise. On the other hand, non-IgE mediated allergy has a delayed onset from one to 24 h after exposure to the allergen, with symptoms affecting the gastrointestinal tract and skin [3,4]. Asthma and food allergy are closely linked. Food allergy has been found to be a potential risk factor for the development of asthma. Food allergy often coexists with asthma, and is associated with increased morbidity/mortality among children and adults who have these conditions. Patients with food allergy and asthma are at a higher risk of fatal anaphylaxis (severe allergic reaction) [5,6,7]. A review that examined the relationship between asthma and food allergies found that wheat, seafood, turkey, legumes, cow’s milk, mustard, corn and peanuts are common food allergies in asthmatic children [7].

The incidence of asthma and allergic diseases is still unclear, but diet is thought to play a significant role [8]. The Western diet, characterized by a high intake of sweets, high-fat dairy products, refined grains, fried foods and red/processed meats, has pro-inflammatory effects, whereas the Mediterranean diet, characterized by a high intake of whole grain cereals, seafood, fruits and vegetables, has demonstrated anti-inflammatory and antioxidant properties [9]. Fish and fish oil, which are good sources of long-chain polyunsaturated fatty acids, may be beneficial in reducing the risk of severe asthma in children and pregnant/lactating women [10]. In children, dietary vitamin D intake was found to be inversely associated with the risk of asthma [11], but not with food allergies [12].

The AGR consists of six countries, namely Qatar, Saudi Arabia, Bahrain, Oman, Kuwait and the United Arab Emirates (UAE), that together make up the Gulf Cooperation Council (GCC). Asthma constitutes a growing problem and a substantial burden on health services in the region [13]. The prevalence of asthma has shown great variation across countries. According to a recent systematic review, Saudi Arabia has the highest prevalence of childhood asthma (23%), followed by Oman (20.7%), Qatar (19.8%), Kuwait (16.8%) and UAE (13.6%) [14]. Data from a cross-sectional, observational, population-based study of five Middle Eastern countries reported the highest prevalence of asthma in adults (aged ≥ 18 years) in the Gulf cluster (UAE, Saudi Arabia, Kuwait) (7.6%), followed by Egypt (6.7%) and Turkey (4.4%). Across the Gulf cluster, the highest prevalence was documented in Kuwait (9.5%) and Saudi Arabia (8.3%), followed by UAE (4.9%) [15]. A recent meta-analysis of asthma and Chronic Obstructive Pulmonary Disease (COPD) concluded that Kuwait (25.9%), Qatar (19.8%) and Saudi Arabia (17.6%) had the highest prevalence of childhood asthma in the Eastern Mediterranean region [16].

The high prevalence of asthma in the AGR highlights the urgent need for a better understanding of the major risk factors for asthma. The problem of asthma is linked to exposure to environmental and genetic factors. A recent systematic review has shown that genetic predisposition (family history of asthma), tobacco smoking, geographical area, dust, pets, industrial pollutants, low levels of household income and high levels of stress are the main contributors to asthma in Saudi Arabia [17]. Having a parent with a history of asthma, pet ownership, respiratory illness and parental smoking increased the risk of asthma in Qatari schoolchildren [18,19]. In Kuwait, having a parental history of allergy, pet exposure and tobacco smoke were associated with increased asthma risk in young adults aged 18–26 years [20]. A family history of asthma was found to be associated with a high risk of asthma in UAE adolescents (aged 13–19 years) and their parents [21]. The high prevalence of asthma could also be due to climactic conditions. Sandstorms are common meteorological hazards in desert regions, and the Arabian Peninsula countries are particularly prone to these. Saudi Arabia, Kuwait, UAE, Qatar, Iraq and Sudan have been categorized as the second largest dust source regions worldwide. This phenomenon could have persistent adverse consequences on respiratory health, by increasing the prevalence of asthma in the region. High temperature and humidity in the region may also result in increased pollen and fungal spore concentrations which increase the risk of asthma and allergic respiratory diseases [22].

The high prevalence of asthma in the AGR may also be attributable to the consumption of energy-dense foods that are typical of a Western dietary pattern [23]. Studies investigating the effects or association of diet/nutrients and food allergy with the risk of asthma in four Gulf countries (Kuwait, Qatar, UAE and Saudi Arabia) were mostly cross-sectional and case-control. To date, there is no clear evidence that diet and/or food allergy are associated with asthma in Bahrain and Oman. The reason for focusing on evidence of the association between diet and asthma is that the AGR has recently experienced major shifts in food consumption patterns, such as a large increase in the consumption of animal fats, vegetable oils, sugar and sweeteners [24]. Empirical knowledge on the relationship between food allergy and asthma in the AGR is limited. There is much concern about how “allergic march” and “allergic multimorbidity” concepts explain the interrelationships between allergic diseases. The allergic march concept suggests the temporal progression to asthma comes mainly from atopic dermatitis (eczema) [25]. The concept contends that the coexistence of atopic dermatitis and food allergy especially in those with early-onset and severe eczema heralds the development of other allergic conditions such as asthma [26]. The existing literature is still controversial on whether early life food sensitization is the first step towards food allergy, which may lead to asthma and other allergic conditions. Although a systematic and meta-analysis of cohort studies found that sensitization to food in the first two years was associated with an increased risk of childhood allergic conditions (allergic rhinitis, eczema and wheeze/asthma), these studies did not address this association in later childhood/adolescence and may be unable to rule out confounding variables [27]. It remains unclear whether the coexistence of atopic dermatitis and food allergy manifested by other allergic conditions predict asthma or if it is related to food allergy itself. It is also unclear whether the progression from atopic dermatitis to asthma is causal or a result of interacting genetic/environmental factors [25]. In contrast, allergic multimorbidity refers to the coexistence of two or more allergic conditions such as asthma, rhinitis and eczema developed throughout childhood [28]. Since food allergy is referred to as a “second wave” of allergic conditions [29], understanding the impact of food allergy in the development of asthma in the AGR is needed. Few studies exist on the association between food allergy and asthma in the AGR, and at present the association remains controversial due to the confounding influence of other potential causes such as genetic predisposition of allergy, exposure to pets and obesity. The aim of this commentary is to summarize existing evidence highlighting the effects of diet/food allergy on asthma risk in the AGR.

## 2. Arabian Gulf Countries

### 2.1. Saudi Arabia

The diets of Saudi children and adults are usually characterized by low micronutrient content such as selenium, potassium, calcium, manganese, phosphorus, folic acid, biotin and vitamin E and D [30,31]. Vitamin D deficiency is a major health concern and a highly prevalent condition among Saudi children and adolescents [32]. Only a few studies have assessed food allergy prevalence and/or the effects of diet/nutrients on asthma risk, most of which have been cross-sectional and case-control. A previous cross-sectional study found that the prevalence of allergies to peanuts, egg white and cow’s milk were 22.6%, 14.5% and 12.9% respectively among 217 adults with asthma, urticaria and rhinitis attending King Khalid University Hospital in Riyadh city [33]. Another cross-sectional study aimed at examining the role of clinical sensitivity to eleven food allergies (cow’s milk, nuts, strawberries, fish, tomatoes, bananas, eggs, beer, mushrooms, melon and citrus fruit) was carried out on 1341 children and adults with bronchial asthma attending King Abdulaziz University Hospital. The study found that the prevalence of clinical sensitivity to food allergies was 29%, and patients with a positive family history of allergies and with high total IgE levels have high rates of food allergies [34]. A recent cross-sectional study found that vegetable and egg intake is the main dietary risk factor for the prevalence of bronchial asthma among schoolchildren in Najran city. On the other hand, children who had diets high in fruit, seafood and dairy products had a lower risk of asthma. Results also found that other risk factors such as having dogs at home, using wood fuel for cooking and exposure to traffic were associated with asthma prevalence [35].

In a case-control study conducted on 114 asthmatic and 202 non-asthmatic children aged 12 y in urban and rural areas, asthmatic children reported greater consumption of food from fast food outlets and consumed diets lower in vegetables, milk, magnesium, vitamin E, fiber, potassium, sodium and calcium than their non-asthmatic counterparts [36]. Another case-control study carried out on 70 children aged 4–18 y with and without bronchial asthma attending King Abdul Aziz University Hospital in Jeddah city, found that children with asthma have lower lung function and serum 25(OH)D concentrations (<50 nmol/L), and higher serum levels of atopy markers than children without asthma [37]. A recent case-control study examined the differences of serum 25(OH)D concentrations in adults aged 22–28 years (359 asthmatic, 711 non-asthmatic) in Riyadh city, and found that after adjusting for age, gender and BMI, asthmatic, particularly females, had lower serum 25(OH)D concentrations (<25 nmol/L) than their non-asthmatic counterparts [38]. 

### 2.2. Qatar

According to the 2012 Qatar National STEPwise Survey, most adults (83%) did not adhere to whole grains, legumes, fiber and fruit and vegetable consumption recommendations, and more than half (50–72%) and less than half (47%) reported frequent consumption of sweets/beverages and fast foods respectively [39]. There have been only a few studies examining the associations between diet/nutrients and/or breastfeeding with the risk of asthma. A cross-sectional study examined the associations between breastfeeding and risk of asthma/allergic diseases among 1500 Qatari children (aged < 5 years) recruited from primary health care centres and Hamad hospital, and found that exclusive breast feeding prolonged for >6 months was associated with a reduced risk of asthma, wheezing, eczema and allergic rhinitis. Results also showed that parental history of allergic rhinitis, maternal asthma and mothers having a first baby were the main factors associated with the mode of feeding [40]. A recent cross-sectional study carried out on 986 Qatari adults examined the associations between asthma incidence and soft drink intake and found that after controlling for demographic variables, BMI, inflammation, intake of fruit and vegetable, physical activity and smoking, adults who consumed soft drinks ≥7 times/week were more than twice as likely to have asthma compared to non-consumers [41]. In a case-control study that examined the differences of serum 25(OH)D concentrations in children aged <16 y (483 asthmatic, 483 healthy control) conducted in the paediatric primary care clinics in Qatar, asthmatic children had lower serum 25(OH)D concentrations (<20 ng/mL) than healthy children. Results also showed that asthmatic children had less exposure to sunlight and lower levels of physical activity than non-asthmatic controls [42].

### 2.3. Kuwait

According to the 2009–2010 Kuwaiti National survey data, most Kuwaitis (78–100%), regardless of age, exceeded the recommended protein and carbohydrate requirements, and more than two-thirds of males aged ≥4 years exceeded the recommended upper limit (UL) of sodium. However, less than a half of Kuwaitis met the recommended level for fiber, n-3 and n-6 fatty acids, calcium, folate, and vitamins D and E [43]. The evidence on the effects of food allergy on the risk of asthma is mostly limited to studies on children. In a case-control study, 100 children aged <13 years with food allergies were recruited from pediatric allergy and immunology clinics in Kuwait. They were matched to 100 controls with other types of atopic diseases (asthma, rhinitis, eczema). The study found that cow’s milk was the most common food allergen, followed by eggs, tree nuts and peanuts. Compared to the controls, children with food allergies were less likely to be breastfed for ≥6 months, given a combination of breast milk/formula, adhered to complementary feeding at <6 moths, and were more likely to have asthma and eczema [44]. One recent cross-sectional study carried out on 3864 schoolchildren aged 11–14 years old showed significant associations between food allergy (fish, shellfish, eggs, peanuts or tree nuts) and the development of eczema, rhinitis and asthma, suggesting that food allergy may be considered a risk factor for developing asthma and contribute to its severity [45].

### 2.4. UAE

According to a nationally representative cross-sectional survey of 628 households in seven Emirates, more than one-third of children (38% of boys and 43% of girls) consumed more calories than their average estimates. Beverages (cola, orange squash and root beer) contributed 8% and 14% of the total energy intake for adult women (aged ≥ 19 years) and male children (aged < 10 years) respectively [46]. A recent review reported that 8.1% of children have food allergies, and fish, eggs and fruit were the most common food allergens [14]. The prevalence of food allergy among university students was 9.4%, and the most common allergens were seafood and nuts [47]. Vitamin D deficiency is one of the potential causes of childhood asthma in UAE [48]. There is limited evidence linking food allergy to asthma in UAE. In a previous cross-sectional study, the eight most common food allergens (fish, fruits, eggs, wheat, vegetables, tree nuts, peanuts, and cow’s milk) in 660 schoolchildren in Al-Ain city were found to be associated with a paternal history of asthma, atopic dermatitis and allergic rhino-conjunctivitis and a personal history of atopic dermatitis [49].

## 3. Conclusions

Asthma is a serious health problem affecting both children and adults globally [50]. Unhealthy diets and food allergies play a role in the development of asthma [6,51]. A typical Western diet has been reported to promote inflammation and chronic inflammatory diseases [9]. On the other hand, the Mediterranean diet includes a variety of healthy foods with anti-inflammatory properties that might help to reduce the risk of inflammatory diseases [9].

Current asthma prevalence among children and adults in the AGR has been rising, suggesting that asthma has become a significant public health concern. Despite limited studies, this commentary demonstrates that unhealthy diets and food allergies are significant risk factors for asthma. High intake of soft drink was associated with increased asthma risk in Qatari adults. One explanation could be what the findings also indicated, that obesity and inflammation partly mediated the relationship between soft drink and asthma, which could contribute to an increased risk of asthma. There is evidence indicating that soft drink consumption is related to obesity, inflammation and asthma [52]. Consumption of fast foods, vegetables and eggs was linked to asthma, while consumption of seafood, dairy products and fruit intake were found to be protective in Saudi children. The possible explanations for these apparently contradictory results could relate to different food preparation methods, lifestyle/environmental risk factors, genetic predisposition or the country’s rapid urbanization. Serum vitamin D concentrations were found to be lower in asthmatic children and adults than their non-asthmatic counterparts in Saudi Arabia and Qatar. One explanation for this is that inadequate sunlight exposure and a lack of exercise may contribute to vitamin D deficiency in the Qatari population, resulting in a predisposition to develop asthma. On the other hand, the issue of missing potential confounding factors such as sunlight exposure, physical activity and obesity make it difficult to establish clear conclusions in the Saudi Arabian population.

Allergies to fish, shellfish, eggs, cow’s milk, fruit, vegetables, peanuts and tree nuts were found to be associated with asthma in Kuwaiti and Emeriti children. The proposed explanation for this is that a family history of allergic diseases and food allergy, exposure to pets and obesity are contributing risk factors for childhood food allergy, which could have an impact on increasing the severity of asthma in these populations.

Based on this commentary, further studies are needed to assess the effects of food allergy, single foods and consumption of vitamins and minerals (from both diet and supplements) on asthma severity in children, adults, during pregnancy and lactation, addressing whether such effects have clinical benefits. In particular, randomized control trial (RCT) studies in asthmatic patients are needed to assess whether dietary/supplement interventions could improve asthma symptoms. Several reviews of observational studies and a few RCTs have reported the protective effect of fish/LCn3PUF intake [10], antioxidants from diet (vitamins A, C and E, β-carotene) [53,54] and dietary vitamin D intake [53,55,56] against asthma in children, adults and pregnant/lactating women. However, some trial studies suggest that supplementation with antioxidant vitamins and methyl donor nutrients (vitamin B_6_ and B_12_, folate and choline) did not result in improved asthma outcomes or lung function in asthmatic patients [54]. Further studies are also needed to assess the effects of dietary/antioxidant supplements on improving asthma control in obese patients with asthma. A recent systematic review of RCT studies has shown that magnesium, vitamin C and fatty acid supplementation were beneficial for improving lung function and asthma control in obese adults with asthma [57].

Further studies focusing on strategies for reducing asthma morbidity from a wider dietary perspective are needed to ensure a significant improvement in quality of life for asthmatic patients. Dietary modification through healthy foods could be considered a potential strategy for improving asthma. Antioxidant-rich dietary interventions could have a positive effect on improving asthma control or lung function in children and adults [53]. One RCT suggested that promoting a healthy diet rich in unsaturated fat was feasible in adults with asthma [58]. Behavioural interventions targeting action planning, self-monitoring and problem solving could also be considered an effective strategy to change eating behaviours in favour of healthier foods among adults with uncontrolled asthma [59]. This commentary suggests further interventions should be undertaken targeting these behaviours in reducing consumption of a Westernized dietary pattern in asthmatic children and adults. There is a critical need for future interventions targeting poor diet, weight loss and physical inactivity, which could be seen as negative health consequences for asthma [60]. Future studies are also needed to develop food-based dietary guidelines to provide nutrition education and reduce the risk of asthma in the AGR. There are no clear dietary recommendations for asthma prevention in the AGR. However, scientific evidence has led to the development of food-based dietary guidelines to minimize and hopefully prevent diet-related diseases in the region. The guidelines include directions to (1) eat an adequate daily intake of fruit, vegetables, cereals and their products, and milk and dairy products; (2) eat chicken, fish, meat, nuts and legumes on a regular basis; (3) reduce the intake of foods with a high content of fat, sugar and salt; (4) drink enough water daily; and (5) avoid drinking alcohol [61]. There is strong evidence supporting the benefits of healthy diet and specific vitamin supplements for asthma control in children and adults (Table 1). This may benefit patients with asthma in the AGR.

The management of food allergy requires careful monitoring and accurate diagnostic evaluation to ensure asthmatic children and adults are consuming nutritionally balanced diets, and excluding offending foods that can cause severe reactions [62]. Further studies should focus on using reliable tools for diagnosing food allergies. Tools used for diagnosing food allergies have received less attention in terms of their effectiveness. According to a systematic review and meta-analysis study, specific-IgE and skin prick tests have good sensitivity but poor specificity for the diagnosis of IgE-mediated food allergies. The sensitivity order for both tests was peanuts, egg, cows’ milk, wheat and soy [63]. Dietitians should be actively involved in the diagnosis and management of food allergy in order to improve the quality of life and nutritional status of food allergic patients [64]. Patients at high risk of developing food allergies should be advised to avoid foods that can trigger allergic reactions, so they can make sure that food is safe to eat.

## Figures and Tables

**Table 1:** The effects of food intake/dietary patterns and supplements on asthma control ^a^.

Diet/Supplements	Childhood	Adulthood
Mediterranean diet	Beneficial effect	No effect
Fruit	Beneficial effect	Beneficial effect
Vegetables	Beneficial effect	Beneficial effect
Vitamin C supplement	Beneficial effect	No effect
Vitamin D supplement	Beneficial effect	No effect

^a^ Table derived from Reference [9].
